# Edible Bioactive Film with Curcumin: A Potential “Functional” Packaging?

**DOI:** 10.3390/ijms23105638

**Published:** 2022-05-18

**Authors:** Josemar Gonçalves Oliveira Filho, Mariana Buranelo Egea

**Affiliations:** 1 School of Pharmaceutical Sciences, São Paulo State University (UNESP), Araraquara 13560-570, São Paulo, Brazil; josemar.gooliver@gmail.com; 2 Goiano Federal Institute of Education, Science and Technology, Campus Rio Verde, Rio Verde 75901-970, Goiás, Brazil

**Keywords:** turmeric, bioactive compounds, edible films, functional foods

## Abstract

Edible packaging has been developed as a biodegradable and non-toxic alternative to traditional petroleum-based food packaging. Biopolymeric edible films, in addition to their passive protective function, may also play a bioactive role as vehicles for bioactive compounds of importance to human health. In recent years, a new generation of edible food packaging has been developed to incorporate ingredients with functional potential that have beneficial effects on consumer health. Curcumin, a bioactive compound widely used as a natural dye obtained from turmeric rhizomes (*Curcuma longa* L.), has a broad spectrum of beneficial properties for human health, such as anti-inflammatory, anti-hypertensive, antioxidant, anti-cancer, and other activities. To demonstrate these properties, curcumin has been explored as a bioactive agent for the development of bioactive packaging, which can be referred to as functional packaging and used in food. The aim of this review was to describe the current and potential research on the development of functional-edible-films incorporating curcumin for applications such as food packaging.

## 1. Introduction

Packaging plays an important role in food containment and preservation. However, the materials used in their manufacture, especially those derived from petroleum, are difficult to recycle and are considered non-biodegradable, which results in a negative impact on the environment, with increased levels of pollution and environmental imbalance [1]. To minimize the negative impact of food packaging on the environment, it is necessary, in addition to advances in the development of more efficient methods for recycling these products, to identify new alternatives for sustainable and/or biodegradable packaging materials that are simultaneously efficient for food protection and have minimal or no negative environmental impact [2].

Edible and/or biodegradable biopolymer films as biodegradable and non-toxic alternatives to petroleum-derived polymers have stimulated interest in the scientific community [1,3]. Edible films are thin layers made from food-borne materials capable of providing a barrier against gases, moisture, and the movement of solutes in food. Edible film materials are potential substitutes for traditional petroleum-based packaging materials due to their excellent properties, such as biocompatibility, edibility, and wide range of applications [4]. Edible films are made from organic macromolecules, usually proteins and polysaccharides, or a mixture of both and a plasticizer [5]. Plasticizers are added to edible films to improve the flexibility and processability of the polymers, lowering the second-order transition temperature and the glass transition temperature [6].

Edible films for food, in addition to their passive protective function, may also play a bioactive role in transporting compounds of interest to human health. In recent years, a new generation of edible food packaging has been developed to incorporate functional ingredients that can have beneficial health effects on consumers [7,8].

There is a wide diversity of biomolecules, known as bioactive compounds, present in plants with important biological properties, which include antihypertensive, antioxidant, antimicrobial, anti-obesogenic, anti-inflammatory, and anticancer activities, among many others. These bioactive compounds can be classified into polyphenols, terpenoids and polyenes, nitrogenous phytochemicals, and organosulfur compounds. In nature, the most abundant group are polyphenols, including flavones, flavonoids, isoflavones, catechins, tannins, curcuminoids, and others [9]. The most common plant bioactive compounds, which are also pigments, in vegetables and fruits are betalains, chlorophylls, curcumin, anthocyanins, and carotenoids. In addition to their coloring properties, these plant pigments exhibit potential health-promoting functions [10].

Flavonoids, in addition to their important role in the secondary metabolism of plants, are fundamental components in the human diet due to their multiple effects on human health, such as lower risk of non-fatal infarction and ischemic stroke [11], beneficial effects for the cardiovascular system [12], as well as vascular and cerebrovascular health [13]. Curcumin is a flavonoid traditionally used in the food industry for flavoring and color because of its characteristic aroma and yellowish-orange appearance [14,15]. In addition to these properties, curcumin has great pharmacological potential as an antioxidant [16], antibacterial [17], antiviral, anti-inflammatory, anticancer, and antidiabetic agents; it is also being used in the treatment of cardiovascular diseases, metabolic syndrome, arthritis, and others [18,19,20,21,22].

The application of curcumin as an antioxidant and antimicrobial agent, as well as colorimetric indicator for the development of active and smart biodegradable biopolymer-based films for food, has been extensively explored in recent years and has been reviewed in some recent studies [23,24,25,26,27,28]. The growing interest in the use of curcumin as a bioactive agent in bioactive films, which in this paper will be referred to as functional packaging, occurs because of its functional potential in human health and has driven research into these materials and compounds in recent years. In this context, the aim of this review was to describe the current and potential research surrounding the development of functional edible films incorporating curcumin for applications such as functional food packaging.

## 2. Functional-Edible-Films: A New Concept in Food Packaging

Food packaging has several functions, including those associated with containment, marketing, and information. The main function of traditional food packaging is to separate food from the surrounding environment, reducing the interaction with spoilage factors (such as water vapor, light, oxygen, microorganisms, and others), minimizing alterations in the food, ensuring higher stability, and consequently increasing the shelf life of food during storage [29].

Edible films are defined as primary packaging made from edible ingredients obtained as solid laminates and applied to foods [30]. Edible films have been developed for applications such as wrapping food products and acting as a protective layer without nutritional, functional, or sensory appeal. On the other hand, films with such properties may be desirable for applications such as packaging ready-to-eat foods, such as sushi, sandwiches, and frozen pizza, or even as primary packaging for breakfast cereals [31].

Proteins, polysaccharides, lipids, and combinations of these materials are the main biopolymers used for the development of edible films. Polysaccharides and proteins have received more attention because of their special properties, such as their ability to form a good film, relative abundance, and nutritional quality. Polysaccharides, such as alginate, agar, xanthan, carrageenan, guar, pectin, and locust bean gum, have been widely used to produce edible films [32]. Among proteins, zein, gelatin, casein, whey proteins, and soy proteins have been the most studied for the development of edible films for food [29]. Most lipid compounds are not capable of forming films individually, but some waxes and oils, such as carnauba wax and palm oil, can be used in combination with hydrocolloids to create composite films with improved moisture resistance due to the hydrophobic nature of lipids [33,34].

**Figure 1:**
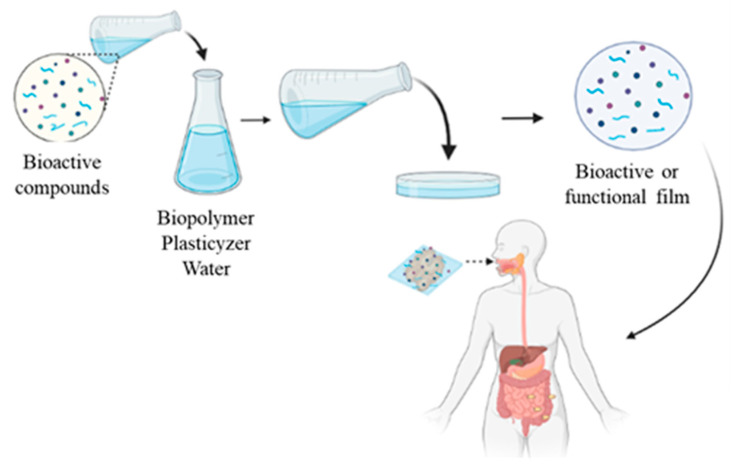
Overview of functional edible films and their actions.

Functional-edible-films with bioactive properties (Figure 1) are designed for consumers who are concerned with nutritional and health aspects, in addition to the sustainability expected for biopolymeric films [35,36]. These packages are edible and contain bioactive substances, such as prebiotics, probiotics, marine oils, and flavonoids [37], which can promote health benefits for consumers [38,39]. Functional-edible-films represent a technology currently emerging as a new strategy, being an innovative approach to the concept of functional foods associated with sustainable approaches [40].

A great advantage of functional-edible-films is that they allow bioactive compounds to be transported stably through the human gastrointestinal tract (GIT) and reach the intestine where they can bind with the intestinal epithelial mucosa and later be translocated to the lymphatic system. Edible films containing bioactive compounds must not release bioactive molecules or disintegrate in the upper part of the GIT, where these molecules are not or are poorly absorbed. Functional-edible-films must be resistant to pH changes and the action of digestive enzymes. In addition, the release of ‘cargo’ molecules must be carefully studied, as the type of polymer used in film development will influence the amount of bioactive agent in the medium, since the material properties depend on the interaction of the biopolymer with the bioactive agent [41].

The term ‘bioactive films’ is mostly used by the scientific community to describe edible films loaded with bioactive compounds capable of promoting beneficial effects for health [7,41,42,43]. However, some studies have used this terminology to describe active films that have beneficial effects on food, such as antimicrobial and antioxidant activities [44,45,46,47,48]. In this review, we propose a new terminology, ‘functional-edible-films’, to describe edible films capable of providing beneficial effects for human health.

## 3. Curcumin: A Bioactive Molecule with Potential for Edible Bioactive Film Production

**Figure 2:**
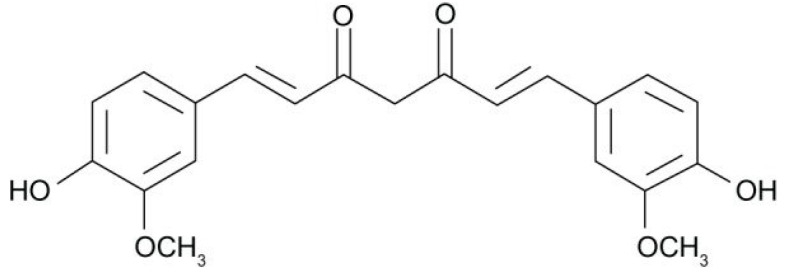
Chemical structure of curcumin.

Bioactive compounds are phytochemicals that can be extracted from food or food byproducts to provide health benefits or modulate metabolic processes [49]. Curcumin is an active ingredient in turmeric that is extracted from the root of turmeric (*Curcuma longa*), belonging to the Zingiberaceae family [50]. Curcumin is a symmetrical molecule characterized by two orthomethoxyphenolic groups connected by a seven-carbon chain and an enol form of β-diketone [51,52] (Figure 2). In addition to its anti-inflammatory, antimicrobial, and antioxidant activities, curcumin has demonstrable beneficial effects on human health, including its use in the treatment of cardiovascular diseases, cancer, metabolic syndrome, diabetes, arthritis, and mental illnesses [18,19,20,53].

Phenolic OH groups present in the structure of curcumin are the main sites that participate in the scavenging of reactive oxygen and nitrogen free radicals and play a key role in antioxidant activity [54,55]. The antioxidant activity of the curcumin molecule improves redox homeostasis, which is directly related to diseases caused by aging [56] through the regulation of nuclear factor erythroid 2-like 2, which promotes hormonal responses and increases defenses against damage caused by oxidants [57]. The antioxidant capacity of curcumin has gained interest in clinical research for the prevention or mitigation of diseases that cause oxidative stress [58].

The effects of curcumin on human health may also be related to its antimicrobial potential [59] that results in a more rapid response in the wound healing process, mainly in elderly patients [60]. Curcumin also demonstrates antiviral activity because it inhibits the enzyme inosine monophosphate dehydrogenase, preventing the oxidation of IMP to xanthosine 5’-monophosphate, and consequently preventing the formation of guanosine 5’-triphosphate, which are fundamental reactions for both DNA and RNA viruses [61]. In both antimicrobial and antiviral activities, the phenolic groups present in curcumin negatively regulate the activation of transcription factors (NF-κB and AP-1) that are associated with inflammatory processes [62].

According to the World Health Organization [63] chronic diseases are responsible for 71% of all deaths globally, including cardiovascular diseases (heart attack and stroke), cancer, and diabetes. For cardiovascular diseases, the mechanism of action of curcuminoids is associated with the regulation of oxidative stress, apoptosis suppression, and anti-inflammatory activity [64]. The anti-inflammatory activity of curcuminoids and the modulation of key molecules, such as kinases, also play a role in inflammation regulation and swelling of joint regions in a disease known as arthritis [65]. Similarly, the anti-obesity activity of curcumin is attributed to anti-inflammatory responses, oxidative stress balance, and endogenous antioxidant enzyme expression [66]. Safarian et al. [67] reported that the ingestion of curcumin (1 g/day) and phospholipidated curcumin complex for six weeks increased the serum zinc and zinc-to-copper ratio, which represent two important antioxidants in patients with metabolic syndrome.

Curcuminoids have also been used to manage diabetes, and the pathways related to this effect involve the release of insulin and glucose metabolism [68,69]. A protective function against diabetes-related health problems was promoted by treating curcumin-supplemented yoghurt to streptozotocin-diabetic rats, decreasing carbohydrate biomarkers by 63% and lipid disturbances (triacylglycerol and total cholesterol in 61% and 21%, respectively) as well as increasing paraoxonase by 31% compared to non-diabetic rats [70]. Likewise, Jamilian et al. [71] reported a reduction in body weight (from 72.1 ± 9.8 to 71.3 ± 9.8 kg), serum lipids (from 163.6 ± 44.8 to 154.0 ± 29.4 mg/dL in triglycerides), and glycemic control (from 111.3 ± 3.6 to 10.1 ± 3.2 µIU/mL), which in turn increases insulin sensitivity in women with polycystic ovary syndrome (n = 60, 26 in placebo group and 24 in curcumin group) after consumption of 500 mg/day of curcumin over 12 weeks.

Curcumin also exhibits anticancer activity through anti-inflammatory activity and other mechanisms, such as suppression of proliferation and apoptosis, cell migration, and invasion by cancer cells, as well as angiogenesis and lymphangiogenesis, which are essential components of the metastatic pathway, and suppresses tumor protein p53 [72].

In addition, these health conditions can increase the chances of developing mental diseases, and mental illness, particularly depression, increases the risk of long-lasting conditions, such as heart diseases and type 2 diabetes [73]. Curcuminoids fit in this context by modulating dopamine and serotonin production, reducing neuroinflammation, regulating oxidative stress, and preserving mitochondrial function to improve mental status [74].

In this sense, curcumin incorporation as a nutraceutical or bioactive agent in commercial food products has attracted interest because of its potential health benefits. However, the main limitations of curcumin are its low solubility in water and poor chemical stability, resulting in low systemic bioavailability and a weak pharmacokinetic profile. Another limitation of using turmeric as a bioactive agent is that high doses of curcumin are usually needed to be effective in vivo [75]. The low bioavailability of curcumin may be the result of a number of physicochemical or physiological processes, including poor solubility and permeability in the GIT, low chemical stability at physiological pH, and rapid metabolization in the GIT and liver [76,77].

## 4. Encapsulation of Curcumin for Application as a Bioactive Agent

**Figure 3:**
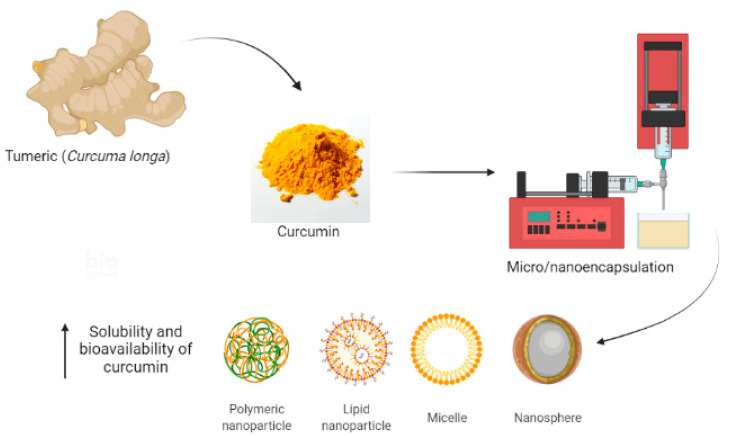
Overview of micro/nanoencapsulation of curcumin.

Curcumin encapsulation in food-grade biopolymers or colloidal delivery systems can overcome the challenges associated with its application [77,78]. Encapsulation technology can increase the solubility and bioavailability of curcumin (Figure 3), while protecting this compound against hydrolysis, enzymatic action, and conjugation inactivation, among others [79,80].

Numerous curcumin delivery systems have been developed, such as micelles, emulsions, nanoemulsions, liposomes, biopolymer nanoparticles, microgels, molecular complexes, and others [77,81,82].

Zheng, Zhang, Peng, and McClements [82] studied the impact of the curcumin delivery system type on bioaccessibility. The authors reported different behaviors in the simulated GIT according to the type of system utilized: nanocrystals, nanoemulsions, and soymilk. The nanocrystal system had the lowest bioaccessibility because there were fewer mixed micelles to solubilize the curcumin molecules. In another study, Peng et al. [83] demonstrated an increase in the bioavailability of curcumin by 2.7–3.6-fold in both in vitro and in vivo studies compared to free curcumin crystals by encapsulating curcumin in sophorolipid-coated nanoparticles.

Liu et al. [84] reported that micelles based on amphiphilic starch and curcumin were able to achieve a sustained release of ~55% curcumin over a 7-h period of intestinal simulation. Gómez-Mascaraque et al. [85] observed that hybrid nanostructures based on phosphatidylcholine liposomes inside a matrix of wood-polymer composite prepared by electrospray for delivery of curcumin were able to increase their bioaccessibility by ~1.7 times compared to the free compound.

Emulsion-based systems have been described as excellent carriers of lipophilic curcumin for improving its stability and bioavailability. Aditya et al. [86] revealed that encapsulation of curcumin in a dual water-in-oil-in-water emulsion increased its bioaccessibility by 4-fold (72%) compared to free curcumin (16%). In another work, Zheng et al. [87] developed curcumin-based nanoemulsions using three different methods: (i) conventional oil loading, (ii) heat driven, and (iii) pH driven. The formulations showed similar bioaccessibility values using the GIT simulator (74–79%), ~7–8 times higher than the curcumin solution (10%). This suggests that encapsulating curcumin in small lipid particles may be advantageous for improving its absorption in the GIT [84].

Studies have revealed that the type of carrier oil used in the formulation of nanoemulsions affects the bioaccessibility of curcumin. Ahmed et al. [88] evaluated the effect of triacylglycerol molecular weight on curcumin bioaccessibility. Emulsions containing only short-chain triacylglycerols within the carrier lipid showed only about 1% bioaccessibility, while emulsions formulated with long-chain triacylglycerols and medium-chain triglycerides showed bioaccessibility of ~40% and ~20%, respectively. The increase in bioaccessibility in emulsions formulated with long-chain triacylglycerols and medium-chain triglycerides is related to the presence of mixed micelles.

Shah, Zhang, Li, and Li [89] reported that curcumin-based nanoemulsions with medium-chain triglycerides had a bioaccessibility of 32%, while nanoemulsions produced with long-chain triacylglycerols had a bioaccessibility of 65%. The bioaccessibility of curcumin may increase with increasing total lipid content due to the increase in mixed micelles available to solubilize curcumin. However, bioaccessibility may not increase when the amount of lipids is higher than a certain content because the lipid phase is not fully digested, which results in an incomplete release of curcumin from the droplets to the mixed micelle phase.

Other approaches have been developed using curcumin loaded into colloidal particles based on the pH shift method [87,90]. Curcumin molecules are deprotonated at pH values < 8.0 and have poor water solubility. However, this molecule is protonated at pH ≥ 12 with high water solubility.

This pH dependence involves the pKa of curcumin to encapsulate the hydrophobic interiors of colloidal particles. Pan, Luo, Gan, Baek, and Zhong [90] used this method to encapsulate curcumin in casein nanoparticles at 0.4 mg/mL, showing an improvement in its anti-proliferative activity using human colorectal (HCT-116) and pancreatic cancer (BxPC3) cells.

The encapsulation of curcumin may be a strategy to enhance its application functional-edible-films for food, contributing to the protection and increase of the bioaccessibility of this compound, contributing to a greater effectiveness related to its beneficial effects on health. Each encapsulation system has advantages and disadvantages depending on the purposes, and many points must be taken into consideration to decide the most suitable according to the application needs. These systems can therefore be used in foods specifically designed to increase the overall oral bioavailability of one or more types of co-ingested bioactive agents.

## 5. Functional-Edible-Films Containing Curcumin

**Figure 4:**
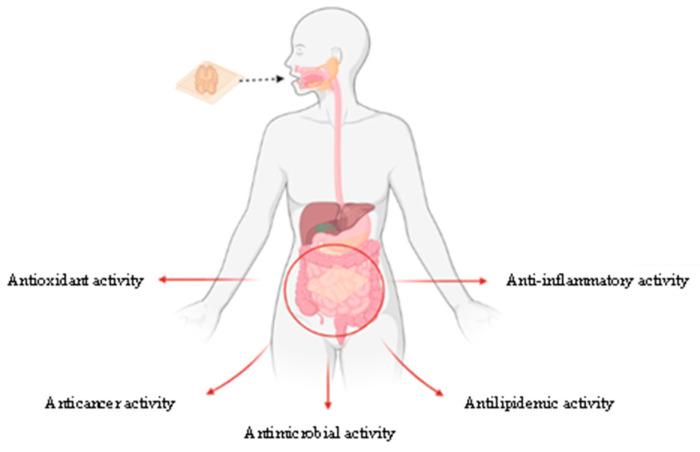
Overview of possible beneficial health effects of bioactive edible films containing curcumin.

Curcumin incorporation as a bioactive agent in functional-edible-films or -coatings has attracted interest because of the potential health benefits (Figure 4). Substantial in vitro studies have been carried out using curcumin in edible films, exhibiting an increase in their antioxidant properties (Table 1) [21,36,44,91,92,93]. Roy and Rhim et al. [44] reported that the incorporation of curcumin in poly(lactic acid)-based films increased the antioxidant activity evaluated by the DPPH and ABTS methods from 1.8% and 3.1 to 76.6% and 94.7%, respectively, by the addition of 1.5% curcumin by weight. Meanwhile, Xiao et al. [94] observed that curcumin addition increased the antioxidant activity of films based on soy protein isolates and cellulose nanocrystals by ~25–35% and ~10–20% in the DPPH and ABTS methods, respectively. Roy and Rhim et al. [93] reported that the addition of 1% curcumin to films based on carboxymethylcellulose and zinc oxide significantly increased the antioxidant activity of the films to 40.2% and 92.5% in the DPPH and ABTS methods, respectively. These studies represent a preliminary means of assessing the health-promoting effects of bioactive edible films developed with curcumin.

In vitro studies have also revealed that edible films based on biopolymers incorporated with curcumin may have antimicrobial activity [95]. Rostami and Esfahani [95] reported that nanocomposite films based on *Melissa officinalis* seed gum films incorporated with montmorillonite and curcumin showed antimicrobial activity against *Escherichia coli*, *Bacillus cereus*, and *Bacillus subtilis*. Taghinia et al. [96] observed that edible films based on *Lalle-mantia iberica* seed mucilage incorporated with curcumin showed antimicrobial activity against *Escherichia coli*, *Bacillus cereus*, *Bacillus subtilis*, and *Penicillium expansum*. Similarly, Manna et al. [97] demonstrated that curcumin-loaded carboxymethylated guar gum can effectively inhibit the growth of gram-positive and gram-negative bacteria. On the other hand, Musso et al. [15] indicated that curcumin-loaded gelatin film (0.02% *w*/*v*) had no antimicrobial effect against *E. coli*, *S. enteritidis*, *B. cereus*, and *S. aureus*.

**Table 1:** Primary effects of curcumin incorporation on the properties of edible bioactive biopolymeric films.

Matrix	Effects/Results	Reference
Poly (lactic acid)	Positively affected: mechanical and UV-barrier properties and antioxidant activity.Negatively affected: contact angle and water vapor permeability.	Akhtar et al. [98]
Soy protein isolate	Positively affected: color and antioxidant activity.	Xiao et al. [88]
Carboxymethyl cellulose	Positively affected: UV-barrier properties and antioxidant activity.Negatively affected: mechanical proprieties and transparency	Roy and Rhim et al. [93]
Poly (lactic acid)/sodium carboxymethyl cellulose	Positively affected: solubility and elongation at breakNegatively affected: tensile strength	Gunathilake et al. [92]
Alginate or carrageenan	Lipid digestion and curcumin release was retarded upon encapsulation.	Zhang et al. [93]
Banana starch	Positively affected: water vapor permeability, and elongation at break.	Sanchez et al. [99]
Gelatin	Positively affected: antioxidant activity.	Musso, Salgado and Mauri [15]
Mucilage of *Melissa officinalis* seed/montmorillonite	Positively affected: antimicrobial activity.	Rostami and Esfahani [95]
Mucilage of *Lallemantia iberica* seed	Positively affected: water vapor permeability, antioxidant, and antimicrobial activities.	Taghinia, Abdolshahi, Sedaghati and Shokrollahi [96]
Carboxymethylated guar gum grafted gelatin	Positively affected: antimicrobial activity.	Manna, Mitra, Pramanik, Kavitha, Gnanamani, and Kundu [97]
Chitosan	Positively affected: yellowness, light barriers, moisture content, water solubility, water vapor permeability, and antioxidant activity.	Rachtanapun et al. [100]
Chitosan nanoparticles	Positively affected: antioxidant activity and inhibit lipid oxidation of fresh pork.	Shen et al. [101]
Cellulose nanofibers/chitosan	Positively affected: edible coating materials were effective in reducing mass loss, firmness loss, respiration rate, and microbial count of the kiwifruits during storage life.	Ghosh et al. [102]
Carboxymethylcellulose	Negatively affected: water vapor permeability and tensile strength.	Bourbon et al. [103]
Alginate	Positively affected: antioxidant activity and inhibit lipid oxidation of fresh pork, beef, and chicken.	Bojorges, Ríos-Corripio, Hernández-Cázares, Hidalgo-Contreras, and Contreras-Oliva [26]

Once curcumin is ingested, several biological and chemical factors slow its delivery to target sites. Problems that limit the effectiveness and usefulness of curcumin include its low bioavailability, which is attributed to its insolubility in water and the rapid metabolism of inactive metabolites [20]. Curcumin is an oil-soluble compound that is practically insoluble at room temperature in water at acidic and neutral pH. Although it is alkaline soluble, it is very susceptible to self-degradation [104]. In the GIT, curcumin binds strongly to mucus, further delaying epithelial cell uptake and subjecting curcumin to auto-oxidation and oxidative degradation [105].

Curcumin-loaded films, as happened in functional-edible-films, can improve the release of this molecule under alkaline conditions owing to the emulsifying capacity of polymers and/or surfactants. The solubility of curcumin increases with increasing concentrations of polymers and/or surfactants in aqueous media. They not only increase the effectiveness but also increase the bioavailability of curcumin by optimally permeating the small intestine and preventing possible degradation in the GIT [106].

The incorporation of curcumin into bioactive edible films can be advantageous in overcoming certain limitations of this molecule, such as low water solubility, low chemical stability, and low bioavailability, which can reduce the in vivo effects [75]. Gunathilake et al. [107] reported that poly(lactic acid)/sodium carboxymethyl cellulose/curcumin films improved the release of curcumin under intestinal pH conditions. The authors showed an increase in curcumin release in phosphate-buffered saline media compared to gastric media due to the better solubility of carboxymethyl cellulose at alkaline pH. Carboxymethyl cellulose is not charged in neutral or alkaline media, swells and solubilizes in phosphate-buffered saline medium, interacts with curcumin, and forms an emulsion, which can enhance the capacity of curcumin to pass through the gastrointestinal mucus layer and be absorbed by epithelial cells. Zhang et al. [108] demonstrated a decrease in curcumin release under simulated gastrointestinal conditions when this molecule was loaded in whey protein microgels, suggesting that biopolymers may prolong curcumin release, rather than increase its bioavailability.

The application of bioactive edible films in food has been investigated in pork, beef, and chicken, as well as kiwifruit [26,101,102]. Ghosh, Nakano, and Katiyar [102] reported that nanofibers/chitosan based cellulose coatings loaded with curcumin were effective in reducing mass loss, firmness loss, respiration rate, and microbial count of the kiwifruits during storage life. In another study, Shen et al. [101] observed that edible coating based on chitosan and curcumin nanoparticles was efficient in reducing lipid oxidation in pork during storage. Bojorges et al. [26] also reported that edible films based on alginate and curcumin were effective in reducing the lipid oxidation of pork, beef, and chicken meat. Despite these promising results, the sensory acceptance, and bioactive effects of ingesting these products have not been investigated in any of the studies.

Curcumin has proven to be an important bioactive agent of natural origin for the development of bioactive edible films for food products due to its biological properties. The use of films as vehicles for curcumin, although it has potential, seems still incipient, as further evaluations are needed in in vivo studies and in food systems. Furthermore, studies related to the bioavailability of curcumin in the chosen vehicle should be included in the research on bioactive films.

## 6. Conclusions

Studies exploring the potential of curcumin in the development of edible bioactive films for foods are preliminary, while studies evaluating the effects of such films in different food systems and in vivo studies employing animal models or humans are lacking. However, some research has been carried out on including curcumins in films to promote beneficial health effects, and curcumin appears to be a promising alternative. This is because curcumin has beneficial effects, and its mechanisms of action have been elucidated. The initial results are encouraging, and as this is an emerging field, studies are expected to increase significantly in the coming years. Future studies should evaluate the effects of bioactive films loaded with curcumin in different food systems and in vivo studies employing animal or human models. Furthermore, the micro- and nanoencapsulation of curcumin should be studied to ensure higher stability of this compound in edible films and during the digestion process.

## Data Availability

Not applicable.
